# Comparative Epigenomics Reveals Host Diversity of the *Trichinella* Epigenomes and Their Effects on Differential Parasitism

**DOI:** 10.3389/fcell.2021.681839

**Published:** 2021-06-11

**Authors:** Yayan Feng, Xiaolei Liu, Yuqi Liu, Bin Tang, Xue Bai, Chen Li, Xuelin Wang, Yiqun Deng, Fei Gao, Mingyuan Liu

**Affiliations:** ^1^Shenzhen Branch, Guangdong Laboratory for Lingnan Modern Agriculture, Genome Analysis Laboratory of the Ministry of Agriculture, Agricultural Genomics Institute at Shenzhen, Chinese Academy of Agricultural Sciences, Shenzhen, China; ^2^Guangdong Provincial Key Laboratory of Protein Function and Regulation in Agricultural Organisms, College of Life Sciences, South China Agricultural University, Guangzhou, China; ^3^Key Laboratory of Zoonosis Research, Ministry of Education, Institute of Zoonosis/College of Veterinary Medicine, Jilin University, Changchun, China; ^4^Comparative Pediatrics and Nutrition, Department of Veterinary and Animal Sciences, Faculty of Health and Medical Sciences, University of Copenhagen, Frederiksberg, Denmark; ^5^Jiangsu Co-innovation Center for Prevention and Control of Important Animal Infectious Diseases and Zoonoses, Yangzhou, Jiangsu, China

**Keywords:** comparative epigenomics, host-related methylomes, hyper-/hypo-methylated SCOs, differential parasitism, *Trichinella*

## Abstract

Comparative epigenomics provides new insights on evolutionary biology in relation with complex interactions between species and their environments. In the present study, we focus on deciphering the conservation and divergence of DNA methylomes during *Trichinella* evolution. Whole-genome bisulfite sequencing and RNA-seq were performed on the two clades of *Trichinella* species, in addition to whole-genome sequencing. We demonstrate that methylation patterns of sing-copy orthologous genes (SCOs) of the 12 *Trichinella* species are host-related and can mirror known phylogenetic relationships. Among these SCOs, we identify a panel of genes exhibiting hyper-/hypo-methylated features in gene-bodies or respective promoters that play pivotal roles in transcriptome regulation. These hyper-/hypo-methylated SCOs are also of functional significance across developmental stages, as they are highly enriched species-specific and stage-specific expressed genes both in Ad and ML stages. We further identify a set of parasitism-related functional genes that exhibit host-related differential methylation and expression among those SCOs, including p53-like transcription factor and Cdc37 that are of functional significance for elucidating differential parasitology between the two clades of *Trichinella*. This comparative epigenome study can help to decipher the environmental effects on differential adaptation and parasitism of the genus *Trichinella*.

## Introduction

Dissecting complex interaction between species and their environments has long been a research hot spot in ecology and evolutionary biology (Duncan et al., 2014). Various factors, such as dietary components, temperature changes, and other external stresses, could modulate the establishment and maintenance of epigenetic modifications, thereby influencing gene expression and phenotype (Feil and Fraga, 2012). As the most studied type of epigenetic modifications found in many taxa, DNA methylation has been confirmed to play a crucial role in transposon silencing, transcriptional regulation, and thus rapid phenotypic variation, which could provide us a deep understanding of how species epigenetically cope with rapidly changing environments (Bender, 2004).

*Trichinella* spp. are the causative agents of human trichinellosis, a parasitic disease that causes substantial morbidity and mortality in billions of animals and humans worldwide, as well as significant losses to the global food production annually (Wang and Cui, 2001; Gottstein et al., 2009; Jiang et al., 2016). All 12 recognized taxa of *Trichinella* spp. identified by molecular means and phylogenetic analyses are genetically and biologically delineated into two distinct clades characterized by the presence or absence of an intramuscular collagen capsule (Zarlenga et al., 2006). Thus, one clade is represented by all species and taxa that accordingly encapsulate in host muscle tissue (*T. spiralis*, T1; *T. nativa*, T2; *T. britovi*, T3; *T. murrelli*, T5; *T. nelsoni*, T7; *T. patagoniensis*, T12; and *Trichinella* genotypes T6, T8, and T9), and the other one does not encapsulate after muscle cell dedifferentiation (*T. pseudospiralis*, T4; *T. papuae*, T10; and *T. zimbabwensis*, T11). Both evolutionary clades share the same life cycles occupying two distinct intracellular niches, intestinal epithelium, and skeletal muscle cells. The muscle larvae (ML) of *Trichinella*, released by host gastric fluids, invade intestine epithelium and subsequently develop into adult worms (Ad). The newborn larvae (NBL) delivered by female adults migrate to skeletal muscles and invade muscle cells where they develop into ML and survive for years (Gottstein et al., 2009). Despite the similarity in life cycles, the two clades showed substantial differences in parasitological, pathological, and immunological characteristics (Wu et al., 2001, 2002; Boonmars et al., 2005). It has been demonstrated that these differences were driven by post-miocene expansion, colonization, and host switching (Zarlenga et al., 2006). Moreover, the two clades also differed significantly from each other in classes of host species. The encapsulated species can only complete their life cycle in mammals by requiring host body temperature ranging from 37 to 40°C, whereas the non-encapsulated species shows a broader host spectrum, including mammals, birds, and reptiles, as it can complete its life cycle at host body temperature ranging from 25 to 42°C (Pozio, 2005). Previous studies have demonstrated that host body temperature had a significant impact on the epidemiology and parasitology of the parasites using experimental and natural infections of reptiles with all of the encapsulated species and non-encapsulated species, *T. pseudospiralis* (Pozio, 2005). Similar results were also obtained in other experimental infections of reptiles belonging to other orders (Loricata, Squamata, and Chelonide) (Pozio et al., 2004). However, experimental infections of birds (e.g., hens, ducks, pigeons, crows, and herons) have shown that these vertebrates are suitable hosts only for non-encapsulated species, *T. pseudospiralis* (Kapel, 2000). Therefore, vertebrate host classes could act as crucial environmental factors to be capable of activating different physiological and immunological mechanisms for parasite development and survival.

Previously, we have characterized DNA methylation profiles in *T. spiralis* and revealed its functional roles on life cycle transition and transcriptome regulation (Gao et al., 2012). Further, we recently documented substantial epigenome differences between the two representative species *T. pseudospiralis* and *T. spiralis*, and confirmed these epigenomic divergences could induce differential interactions at the host-parasite interface (Liu et al., 2021). As a deep coevolutionary association between *Trichinella* species and vertebrate host classes has been investigated using both nuclear small-subunit rDNA (Zarlenga et al., 2006) and protein-coding gene sets (Korhonen et al., 2016), we speculate that epigenetics in relation with host diversity also play a key role in the evolution and adaptation of the 12 *Trichinella* species.

Here, we performed whole-genome, methylome, and transcriptome sequencing on the 12 *Trichinella* species. We observed that methylome divergence of these *Trichinella* species is host-related, and this epigenomic conservation is a regulatory mechanism of transcription. By dividing SCOs into hyper- or hypo-methylated categories, we showed that these two classes of genes exhibiting functional significance on gene expression, especially for hypo-methylated ones, which are related to active transcription. We further revealed that stage-specific expressed genes are also highly enriched in those genes of the two categories. By comparing DNA methylation patterns between the two clades of *Trichinella*, we identified a panel of parasitism-related genes exhibiting differential expression under epigenetic regulation, including p53-like transcription factor and Cdc37, etc.

## Materials and Methods

### Parasite Culture and Collection

Twelve isolates representing all 12 recognized species and genotypes of *Trichinella* were provided by the International Trichinella Reference Center and preserved by serial passage in BALB/c mice in OIE Collaborating Center on Foodborne Parasites in Asian-Pacific Region. The 12 isolates included the following: *T. spiralis* (ISS534), *T. nativa* (ISS70), *T. britovi* (ISS120), *T. pseudospiralis* (ISS13), *T. murrelli* (ISS417), *T. nelsoni* (ISS37), *T. papuae* (ISS1980), *T. zimbabwensis* (ISS1029), and *T. patagoniensis* (ISS2496) as well as *Trichinella* genotypes T6 (ISS34), T8 (ISS124), and T9 (ISS409). Muscle larvae (ML), adult worms (Ad), and newborn larvae (NBL) were recovered as described previously (Liu et al., 2011). Briefly, infectious muscle larvae were harvested by 1% pepsin-HCl digestion of minced muscle tissue from rats 35 days post-infection (dpi). Purified adult worms and newborn larvae were collected from intestines of Wistar rats at 30 h or 6 dpi which were inoculated with each *Trichinella* species with a dose of 8000 larvae per rat. Each sample was snap-frozen in liquid nitrogen and stored at −80°C until nucleic acid isolation. The intestines were then fragmented and further incubated with 0.9% sodium chloride solutions at 37°C for 3 h. Adult worms were harvested by centrifugation. To obtain newborn larvae, adult worms collected at 6 dpi were incubated in Iscove’s Modified Dulbecco’s Medium (IMDM) in 75-cm^2^ cell culture plates at 37°C. The newborn larvae were harvested every 12 h. The animals were treated according to the guidelines of the National Institutes of Health (publication no. 85-23, revised 1996).

### Identification of SCOs in *Trichinella* spp.

The genomic data resources of the 12 *Trichinella* species were downloaded from NCBI BioProject database with the following accession codes: PRJNA12603 for *T. spiralis*, PRJNA451013 for *T. pseudospiralis*, and PRJNA257433 for 10 other *Trichinella* species. OrthoMCL (Fischer et al., 2011) was used to cluster protein sequences of the 12 *Trichinella* species into orthologous groups based on their sequence similarity. As a result, a total of 2708 SCOs among these 12 *Trichinella* species were identified (Supplementary Data 1).

### Whole-Genome Sequencing and Phylogenetic Analysis

High molecular DNA from the 12 *Trichinella* species were extracted using the phenol-chloroform extraction method from freshly collected muscle larvae. Ultraviolet-Vis spectrophotometry with a NanoDrop 2000 (Thermo Fisher Scientific, CA, United States) was used to determine the quantity and quality of total DNA. Paired-end sequencing libraries with an insert size of 300 bp were conducted on HiSeq 2,500 platform, according to instructions provided by Illumina. FastQC (v0.11.7)^1^ was used to assess the quality of sequence reads. Adapters and low-quality reads were removed from raw sequencing reads by Trimmomatic (v0.36) (Bolger et al., 2014) with the following parameters: LEADING:3, TRAILING:3, SLIDINGWINDOW:4:15, MINLEN:75. Clean reads from each species were mapped to the respective reference genome with BWA-MEM algorithm (Li and Durbin, 2010). Approximately 86.5% of these clean reads could be uniquely aligned to the respective reference genome (Supplementary Data 2). The mapping results were converted to bam format by SAMtools (v1.6) (Li et al., 2009). The resulting bam files were sorted by coordinate using SortSam.jar embedded in Picard-tools (v1.118). The flow chart in Supplementary Figure 1 describes the strategies we used to identify single nucleotide variant (SNVs) among the 12 *Trichinella* species. Firstly, we conducted intraspecific single nucleotide polymorphism (SNPs) by Genome Analysis Tool Kit (GATK). Subsequently, we used MUMmer (v3.0) (Kurtz et al., 2004) to examine interspecific SNPs between reference genomes of *T. pseudospiralis* and other 11 *Trichinella* species. Lastly, SNPs from uniquely aligned regions with homozygous genotypes (GT = 1/1) constituted the final set of SNVs. In total, we obtained 5,395,250 common SNVs across the 12 *Trichinella* species. FastTreeMP^2^ was used to construct a phylogenetic tree of the 12 *Trichinella* species under GTR+CAT model based on the concatenated common SNVs.

### WGBS Library Construction, Sequencing, and Data Analysis

Genomic DNA were extracted using phenol-chloroform extraction method. The quantity and quality of DNA were tested by ultraviolet-Vis spectrophotometry with a NanoDrop 2000 (Thermo Fisher Scientific, CA, United States). Genomic DNA from the three life stages (Ad, ML, and NBL) of all *Trichinella* species were selected for WGBS library construction as previously described (Jeong et al., 2016). Briefly, approx. two to five micrograms of genomic DNA were first fragmented by Covaris to an average size of 200–300 bp. End-repair, A-tailing, and ligation were conducted as the Illumina PE genomic DNA sample prep kit protocol, except that ligation was performed using methylated cytosine PE adapters provided by Illumina previously (Jeong et al., 2016). Ligation products were purified and bisulfite converted using the EZ DNA Methylation-Gold Kit (ZYMO) according to the manufacturer’s instructions. The libraries were sequenced using Illumina HiSeq 2,500 platform.

After base calling, raw sequencing reads with more than 30% “N”s in over 10% of the reads or smaller than 30 bp were discarded. Clean reads were aligned to the respective reference genome using BSMAP (v2.73) with a maximal four mismatches and gap size up to 2 nucleotides (Xi and Li, 2009). The methylation level of each cytosine was calculated as the number of reads ascertained methylated to the total number of reads for each covered CpG site. Density methylation of any genomic region was measured by the number of methylated CpGs to the total number of CpG sites. To accurately investigate methylation levels in each species, we used CpG sites with a minimal 5× sequencing depth per strand. In total, an average of 33 million reads was generated for each species, among which 84.28% could be uniquely aligned to the respective reference genome after quality control, reaching an average read depth of 19.55× at the covered methylated sites. The mean bisulfite conversion rate of C to T was up to 99.48% (Supplementary Data 2).

### RNA-Seq Library Construction, Sequencing, and Data Analysis

Total RNA of the three life stages (Ad, NBL, and ML) of all *Trichinella* species were extracted using Trizol reagent (Invitrogen, CA, United States) according to the manufacturer’s instructions. Paired-end sequencing libraries with an insert size of 300 bp were conducted on the HiSeq 2,500 platform following Illumina’s protocols. SOAPnuke (Chen et al., 2018) was used for trimming adapters and filtering on length and quality of the raw sequencing reads, with the following parameters: -q 0.5 -d -Q 2 –sanger -l 20. Clean reads were mapped to the respective reference genome using hisat2 (Kim et al., 2015) with default settings. The gene expression levels were quantified using Cufflinks (v2.2.1) (Trapnell et al., 2012) with reads per kilobase million (RPKM). We obtained an average of 41 million raw reads for the three life stages of each species. After quality control and removal of duplication, approximately 66.59% could be uniquely mapped to the respective reference genome. Of total predicted genes in the respective reference genome, the expression levels of 9,060 genes (82.95%) were quantified based on sequenced reads, averagely (Supplementary Data 2).

We adopted the following approaches to identify stage-specific expressed genes for each of these *Trichinella* species. Firstly, differentially expressed genes between any two developmental stages were identified using DESeq2 (Love et al., 2014). Subsequently, genes exhibiting differential expression both in Ad and NBL stages when comparing with ML stage, whereas differential expression was not observed between Ad and NBL stages, were identified as stage-specifically expressed genes by ML stage (multiple-testing corrected *P* ≤ 0.05). The same forward approach was applied to identify genes that were specifically expressed by Ad and NBL stages.

### Quantifying Evolutionary Changes of DNA Methylation Levels, Gene Expression Levels, and Genomic Sequences

To investigate the evolutionary associations among DNA methylation levels, gene expression levels, and genomic sequences, we examined multiple alignments of the SCOs between *T. pseudospiralis* and 10 other *Trichinella* species using mafft (v7.158b) with automatic detection strategy (except for *T. spiralis*). Then we quantified the epigenetic conservation level using the *P*-values generated by comparing the common mCGs of orthologous genes between the two species under consideration. In each comparison, the expression level of every gene was regressed to the DNA methylation level of the upstream 2-Kb region of the gene. The predicted interspecies gene expression differences were regressed to the epigenomic differences. Sequence differences were estimated using blastn score differences by comparing differences between the maximum log blastn score of all orthologous promoters (4,000 bp centered at transcriptional start sites) and the blastn score of the orthologous promoter pair under consideration (Xiao et al., 2012).

### Classification of SCOs Into Hyper- and Hypo-Methylation Categories

We used two different approaches to distinguish genes into high and low methylation categories following the method described in Sarda et al. (2012). We first used the observed vs. expected proportion of CpG dinucleotides (CpG_O/E_), which could assess the depletion of CpG dinucleotides for genomic regions of interest. Briefly, this measurement is described as:

(1) $${\text{CpG}}_{O/E}=\frac{{\text{P}}_{\text{CpG}}}{{\text{P}}_{\text{C}}\times {\text{P}}_{\text{G}}}$$

where P_CpG_, P_C_, and P_G_ represent the frequencies of CpG dinucleotides, C nucleotides, and G nucleotides, respectively. Genes with a CpG_O/E_ value lower than 0.8 were classified as hyper-methylated genes, whereas genes with CpG_O/E_ greater than 1.0 were classified as hypo-methylated genes. In the second approach, we divided genes into high and low categories using fraction methylation levels. If the methylation level of a gene is 0.01 lower than the average methylation level of whole genes, thus the gene could be classified as a low category. Otherwise, the gene could be classified as a high category. The cut-off values of the two approaches were chosen manually, based on density distributions of the respective data. Genes meeting the above two criteria were classified as hyper- or hypo-methylated SCOs. The same forward approach was also applied to promoter regions of the 2708 SCOs.

### Statistical Analysis

Hierarchical clustering analysis was performed using “pvclust” package in R (version 3.6.2) based on fractional methylation levels of gene-bodies or promoters of the 2708 SCOs. Differentially methylated SCOs were identified as follows: Firstly, density methylation of genes or promoters of members of SCOs between the two clades of *Trichinella* species were compared using the Mann-Whitney *U* test. Subsequently, gene-bodies or promoters with Benjamini-Hochberg corrected *P* ≤ 0.05 were identified as differentially methylated SCOs. Functional enrichment analysis was performed using the Database for Annotation, Visualization and Integrated Discovery (DAVID, v6.8) (Sherman and Lempicki, 2009). Linear regression analysis was conducted using “lm” package in R (version 3.6.2). Expression levels of SCOs between different host classes of *Trichinella* species were compared using *t*-test in R (version 3.6.2), and multiple testing-corrected *P* ≤ 0.05 were reported as differentially expressed genes.

## Results

### Phylogenetic Analysis Across *Trichinella* spp.

**FIGURE 1:**
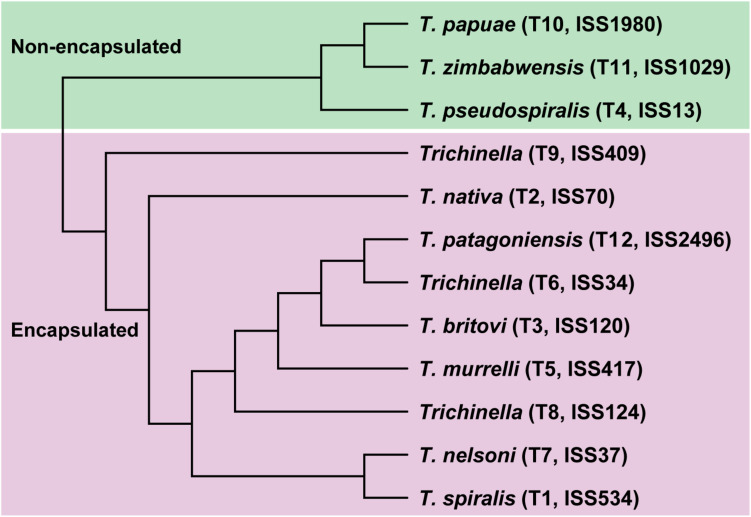
Phylogenetic analysis of the 12 *Trichinella* species based on common SNVs.

In the present study, we applied a multi-omics strategy to thoroughly decipher the molecular characteristics in response to speciation and adaptation in the genus *Trichinella* based on their genetic and geographic diversity. Thus, we generated three types of high-resolution sequencing datasets, including whole-genome sequencing for the ML stage, and whole-genome bisulfite sequencing (WGBS) and RNA-sequencing for the three life stages, for the 12 *Trichinella* species. In total, all of these sequencing reads were well aligned to the respective reference genome after quality control (see section “Materials and Methods” and Supplementary Data 2). Based on the whole-genome sequencing data generated, we obtained high-quality SNV maps/profiles for each of the 12 *Trichinella* species. A consensus tree was constructed by those common SNVs (*N* = 5,395,250) across the 12 *Trichinella* species (see section “Materials and Methods” and Figure 1), which was in agreement with previous phylogenetic analysis using concatenated protein sequence data of other 12 *Trichinella* species in Korhonen et al. (2016), indicating a similar genomic variation of these *Trichinella* species in our dataset.

### DNA Methylation Patterns Across *Trichinella* spp.

Reciprocal best BLAST searches confirmed the orthologous genes encoding the machinery for establishing and maintaining DNA methylation in all 12 *Trichinella* genomes. Phylogenetic analysis further revealed that these three DNMT genes are highly conserved across *Trichinella* spp. (Supplementary Figure 2). RNA-seq analyses indicated these genes of DNA methylation machinery are functionally expressed in all three life stages (Supplementary Figure 3). Accordingly, our data generated via WGBS revealed a similar distribution pattern of genome-wide methylated cytosines for the 12 *Trichinella* species. The highest amount of methylated Cs detected in Ad and ML stages were in the CpG sequence context, whereas NBL stage showed higher numbers of mCHH (H represents A, C, or T). Further, the average percentage of methylated CpG was 10.44 and 10.27% in Ad and ML stages, respectively, whereas NBL stage showed rare traces of DNA methylation (0.46%) (Supplementary Data 2). By profiling methylated CpGs around genic regions, a notable trend was observed in the density of methylated CpG and the distance from the transcriptional start site (TSS), with methylated CpG density increasing toward the transcriptional end sites and returned to the same level as 2-Kb upstream at the 2-Kb downstream (Supplementary Figure 4). Further, exons exhibited higher methylation levels in comparison to introns or flanking regions (Supplementary Figure 5). Similar to plants and mammals, DNA methylation levels of repetitive sequences were significantly higher than those in gene-body regions (Supplementary Figure 6).

### Host-Related Methylome Variation of SCOs Could Reflect Evolutionary History

**FIGURE 2:**
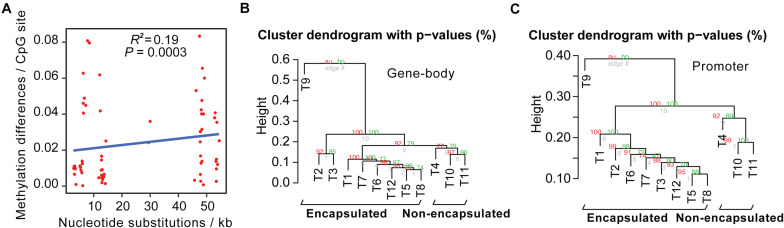
Interspecies methylome variation could reflect evolutionary history across *Trichinella* spp. **(A)** Methylation changes correlate with sequence differences. The *x*-axis represents the number of nucleotide substitutions between the two species per kb. The *y*-axis represents methylation differences of interspecies conservative methylated CpG sites. **(B,C)** Hierarchical clustering analysis based on DNA methylation levels of the gene-bodies **(B)** or promoters **(C)** of the 2708 SCOs in Ad stage.

We then calculated pairwise sequence differences and methylome variations of conservative sites between these 12 *Trichinella* species. Upon comparison of sequence divergence and methylation variation of common CpG sites, we showed that interspecies nucleotide substitution rates exhibited a significantly positive correlation with DNA methylation differences (*P* = 0.0003; Figure 2A).

Next, we detected interspecies methylome divergence of the 12 *Trichinella* species. A hierarchical clustering analysis based on gene-body methylation (gbM) levels of the 2708 SCOs revealed that these 12 *Trichinella* species were clustered into two main clades according to host classes in Ad stage (Figure 2B): (i) species that can only complete their life cycle in mammals requiring a host body temperature ranging from 37 to 40°C, including all of the nine encapsulated species; (ii) species that can complete their life cycle in mammals, reptiles, and birds at host body temperature ranging from 25 to 42°C, including the three non-encapsulated larvae. A similar trend was also observed in DNA methylation levels of promoter regions of these SCOs in Ad stage (Figure 2C), suggesting that promoter sequences of these SCOs also exhibited similar conservative features with that observed in gene-bodies under the strength of evolutionary constraints. An almost identical pattern of hierarchical clustering was achieved for the DNA methylation levels of ML stage both in gene-bodies and promoters among these 12 *Trichinella* species (Supplementary Figure 7). The results of the clustering pattern based on methylation levels are also in concordance with phylogenetic analysis based on common SNVs of the 12 *Trichinella* species (Figure 1), suggesting that DNA methylation divergence of conservative sites might be associated with the evolutionary history of these *Trichinella* species. Further study of the correlation between CpG DNA methylation and proximal genetic variations provided strong support for this observation (Supplementary Figure 8).

### Interspecies Methylome Variation of SCOs Could Be Predictive of Gene Transcription Changes Across *Trichinella* spp.

**FIGURE 3:**
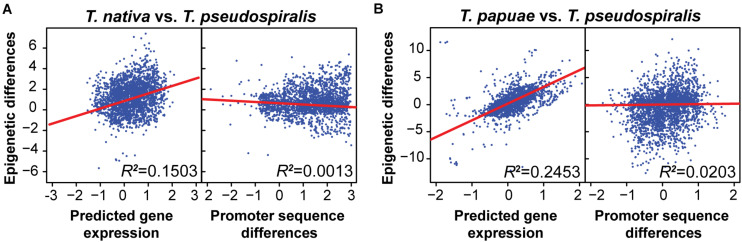
Interspecies methylome conservation is a regulatory mechanism of transcription across *Trichinella* spp. Correlations among DNA methylation changes, gene expression alterations, and sequence differences in *T. nativa* **(A)** and *T. papuae* **(B)** when comparing with reference species *T. pseudospiralis*. Epigenetic differences were computed using the *P*-values generated by comparing the common methylated CpGs of orthologous genes between the two species under consideration. Predicted gene expression differences of every gene were regressed to the DNA methylation level of the upstream 2-Kb region of the gene. Sequence differences were estimated using blastn score differences by comparing differences between the maximum log blastn score of all orthologous promoters (4,000 bp centered at transcriptional start sites) and the blastn score of the orthologous promoter pair under consideration. *R*^2^, square of the sample correlation coefficient.

Previously, we have indicated that interspecies epigenomic changes could be predictive of interspecies changes of gene expression, whereas differences of genomic sequences are not well correlated with gene expression changes (Liu et al., 2021). To test whether this trend is pervasive among the genus *Trichinella*, we compared epigenomic differences to both genomic and transcriptomic changes between reference species *T. pseudospiralis* and 10 other *Trichinella* species. Concordant with our previous findings, here, we observed interspecies epigenomic differences exhibited no clear correlations with sequence differences. However, a much higher correlation was observed between epigenomic and transcriptional changes both in the species of encapsulated (Figure 3A and Supplementary Figure 7) and non-encapsulated clade (Figure 3B and Supplementary Figure 9), suggesting that epigenomic variation can provide a better foundation to evaluate the functional divergence of *Trichinella* species.

### Gene Expression Patterns of Hyper-/Hypo-Methylated SCOs

**FIGURE 4:**
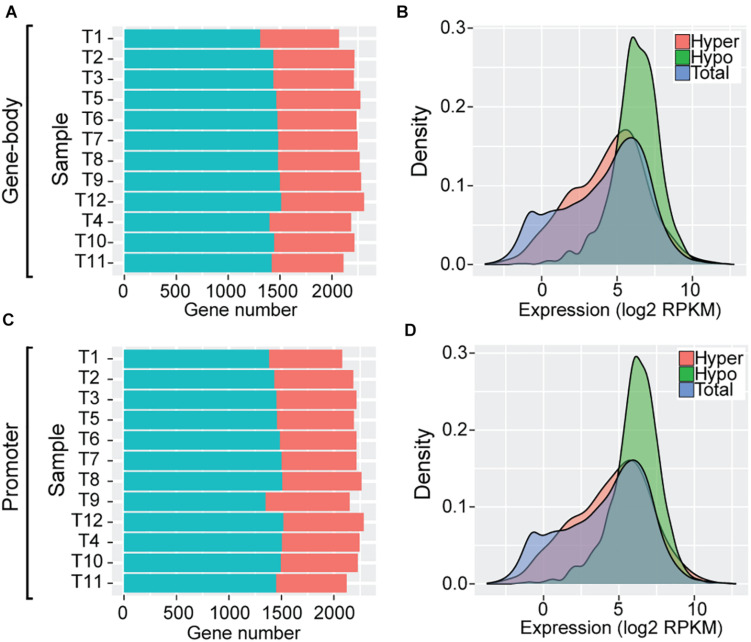
Gene expression patterns of SCOs with hypo/hyper-methylated gene-bodies or promoters of *T. pseudospiralis*. **(A,C)** Numbers of hyper-/hypo-methylated SCOs in gene-body **(A)** and promoter **(C)** regions. **(B,D)** Comparison of expression level density of genes exhibiting hyper-/hypo- features in gene-bodies **(B)** or promoters **(D)** in Ad stage. “Total” represents the number of total genes across the whole genome and is color-coded in blue. “Hyper” and “Hypo” represent the number of genes with hyper- or hypo- methylated SCOs in gene-bodies or corresponding promoters and are color-coded in red and green, respectively.

To further examine the impact of DNA methylation variation on transcriptome regulation of these SCOs (*N* = 2708) during *Trichinella* evolution, we classified these SCOs into high and low categories according to mean methylation levels and CpG_O/E_ values of gene-bodies (section “Materials and Methods”). In total, we identified an average of 774 hyper-methylated (mean = 774 ± 30) and 1,444 hypo-methylated (mean = 1,444 ± 55) genes in Ad stage (Figure 4A). As exemplified by analysis of *T. pseudospiralis*, functional annotation revealed that many biological processes and pathways bear relevance to *Trichinella* parasitism, such as regulation of transcription (GO:0006355, *P* = 6.08E-8), transcription factor activity (GO:0003700, *P* = 1.15E-5), G-protein coupled receptor activity (GO:0004930, *P* = 1.5E-4), and collagen trimer (*P* = 5.6E-4) (Supplementary Data 3). Then we examined the expression patterns of these two categories of genes in comparison to the genome overall. We observed that the hypo-methylated SCOs displayed significantly higher expression levels, whereas hyper-methylated SCOs exhibited similar expression patterns relative to the genome overall in Ad stage (Figure 4B). Next, we adopted a similar approach to analyze the regulatory roles of promoter methylation of these SCOs on mRNA expression levels. Herein, we identified an average of 738 hyper-methylated (mean = 738 ± 40) and 1,459 hypo-methylated (mean = 1,459 ± 47) promoters in Ad stage (Figure 4C). Similarly, a more prominent trend was observed for genes with hypo-methylated promoters to have higher expression levels such that genes with hyper-methylated promoters exhibited extensive expression spectrum similar to the genome overall in Ad stage in *T. pseudospiralis* (Figure 4D). Such expression patterns were also observed in the SCOs with hyper-/hypo-methylated gene-bodies or promoters of 11 other *Trichinella* species (data not shown).

### Functional Significance of Hyper-/Hypo-Methylated SCOs Across Developmental Stages

**FIGURE 5:**
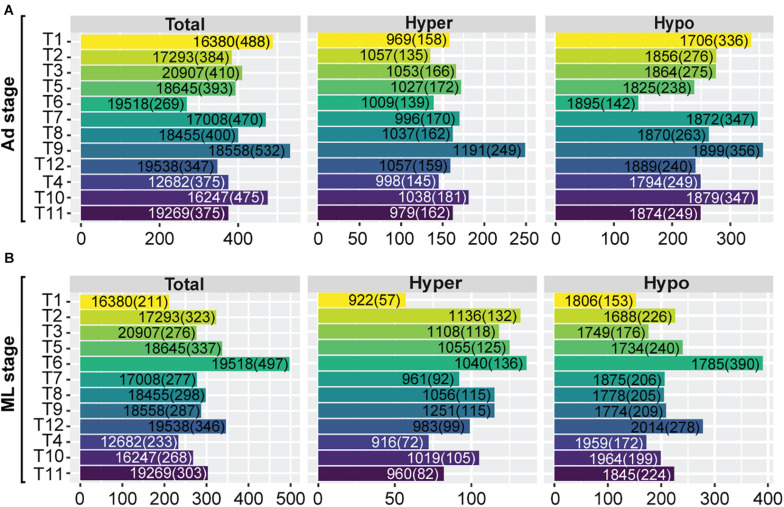
Functional significance of hyper-/hypo-methylated SCOs across developmental stages. Numbers of stage-specific expressed genes of the 12 *Trichinella* species both in Ad **(A)** and ML **(B)** stages. “Total” represents the number of total genes across the whole genome. “Hyper” represents the number of genes with hyper-methylated SCOs in gene-bodies or corresponding promoters. “Hypo” represents the number of genes with hypo-methylated SCOs in gene-bodies or corresponding promoters. Numbers in and outside the brackets represent the number of stage-specific expressed genes and the total number of genes of the respective category, respectively.

We then decipher the functional roles of these hyper-/hypo-methylated SCOs across developmental stages of the 12 *Trichinella* species. As some proteins involved in parasite development and survival against host physiology in *Trichinella* are expressed in a stage-specific manner, we focused on characterizing the stage-specific expressed genes in each of the 12 *Trichinella* species. Based on a stringent criterion (section “Materials and Methods”), an average of 410 Ad stage-specific expressed genes (mean = 410 ± 71) were identified for the 12 *Trichinella* species (Figure 5A), including casein kinase II subunit alpha, putative kinesin motor domain protein, and putative low-density lipoprotein receptor domain class A, etc. The majority of these genes have been previously reported to play crucial roles at the host-parasite interface, parasite survival, and immune evasion in *Trichinella* spp. (Nagano et al., 2009). Then we detected an overlapping trend between these stage-specific expressed genes and the aforementioned SCOs showing hyper-/hypo-methylated features in gene-body or promoter regions. In total, we obtained a panel of 390 non-redundant genes (mean = 390 ± 82), accounting for approximately 95.4% of the total stage-specific expressed genes, might be under hyper-/hypo-methylated gene-body or promoter regulation in Ad stage. Accordingly, a similar proportion (mean = 290 ± 67; 95.2%) of ML stage-specific expressed genes were identified, which contains an average of 304 genes (mean = 304 ± 72) across the 12 *Trichinella* species (Figure 5B). However, these stage-specific expressed genes under hyper-/hypo-methylation regulation are seldomly conserved across the 12 *Trichinella* species, as only three SCOs (fam2462, fam2831, and fam3343) were identified in Ad stage (Supplementary Data 4), suggesting functional divergence of these stage-specific expressed genes in the genus *Trichinella*.

### Functional Significance of Hyper-/Hypo-Methylated SCOs Between the Two Clades of *Trichinella*

**FIGURE 6:**
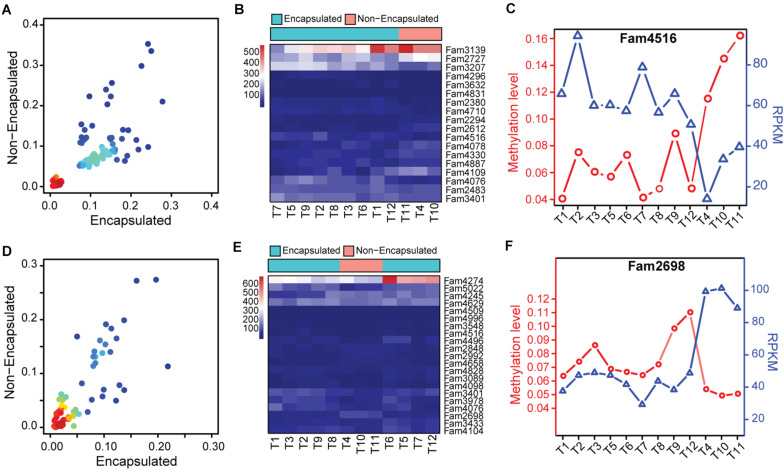
Functional significance of hyper-/hypo-methylated SCOs between the two clades of *Trichinella*. **(A,D)** Scatter plot of differentially methylated genes in Ad **(A)** and ML **(D)** stages, with density at each point (red for high density, blue for low density). **(B,E)** Heatmaps depicting the hierarchical clustering of differentially expressed genes that exhibited differential methylation in gene-bodies or corresponding promoter regions in Ad **(B)** and ML **(E)** stages between the two clades of *Trichinella*. **(C,F)** Methylation and expression levels of fam4516 **(C)** and fam2698 **(F)** of the 12 species.

Based on the methylome variation suggested by the hierarchical clustering analysis, we then focused our analysis on methylation changes of those hyper-/hypo-methylated SCOs between the encapsulated and non-encapsulated clade. Firstly, we merged the SCOs exhibiting hyper-/hypo-methylated gene-body or promoter features together, thus obtained a panel of 1,076 candidate SCOs in Ad stage across the 12 *Trichinella* species. By a cut-off of *P* ≤ 0.05, we identified 139 SCOs that exhibited differentially methylation in gene-body or corresponding promoter regions of these candidates, among which 122 and 17 SCOs exhibited hyper- and hypo-methylation in the encapsulated clade when comparing with the non-encapsulated clade, respectively (Figure 6A). Among these differentially methylated SCOs, 18 of them showed differential expression between the two clades of *Trichinella* (Figure 6B). Thirteen of them have known functional annotations, such as peptidase activity (GO:0008233), peptidase inhibitor (GO:0030414), and gluconeogenesis (GO:0006094) (Supplementary Data 5). Of note, we observed fam4516, encoding an Hsp90 co-chaperone Cdc37, was an Ad stage-specific expressed SCO with significantly higher methylation in gene-body regions, which results in lower expression levels of fam4516 in the three non-encapsulated species (Figure 6C). Moreover, using similar criteria with Ad stage, we identified a total of 87 SCOs that exhibited differentially methylation in ML stage, among which 54 and 33 SCOs showed hyper- and hypo-methylation, respectively (Figure 6D). Among those SCOs, 21 of them showed differential expression between the two clades of *Trichinella* (Figure 6E). Functional annotation revealed that majority of these genes involved in biological processes, such as protein kinase activity (GO:0004672), serine-type peptidase activity (GO:0008236), and ATPase activity (GO:0016887) (Supplementary Data 5). Of note, we observed a non-stage-specific expressed SCO fam2698, a p53-like transcription factor involved in the apoptotic process, exhibited higher expression levels in non-encapsulated clade, which might be modulated by hypo-methylation in its corresponding promoter regions when compared to the encapsulated clade (Figure 6F).

## Discussion

Comparative epigenomics is a powerful tool to understand the conservation and divergence of DNA methylation patterns and regulatory machinery in eukaryotic organisms (Zhong, 2016). For the parasitic nematode *Trichinella*, which possess a complicated life cycle and undergoing a complex developmental regulation of genes, addressing the epigenetic conservation and variation during *Trichinella* evolution may help to understand the differential parasitism and adaptation to different host classes. To date, much of the focus in the genus *Trichinella* has been on the comparison of differential parasitism between the two representative species, *T. spiralis* and *T. pseudospiralis*, using traditional molecular biology (Wu et al., 2001; Boonmars et al., 2005). Studies regarding differential regulation of epigenetic mechanisms across all 12 *Trichinella* species remain largely uncharacterized.

Here we carried out the first comprehensive comparative epigenomic analysis among these *Trichinella* species and revealed that methylome changes in the genus *Trichinella* were host-related, which could recapitulate the known phylogenetic relationships arising from the sequence composition. Paralleled patterns between gbM tree and sequence tree were also observed from other four distantly related invertebrate species, including *C. intestinalis*, *N. vectensis*, *B. mori*, and *A. mellifera*, even though the two trees differ dramatically from the branch length (Sarda et al., 2012). This high interspecies conservation of methylation of orthologous groups implies biological significance, such as assisting acclimatization by modulating gene expression (Takuno and Gaut, 2013; Bewick et al., 2016). That methylation conservation is a regulatory mechanism for transcription is supported by a previous study in which regulatory roles of epigenomic modifications were intensively studied in human, mouse, and pig pluripotent stem cells (Xiao et al., 2012), suggesting that transcriptomes and epigenomes exhibited coevolutionary patterns among those distantly related species.

Relationships between patterns of gbM and transcription regulation in invertebrates have been investigated previously. In insects, genes with high methylation levels tended to be expressed at moderate to high levels. Conversely, a gene with low methylation levels displayed a broad spectrum of mRNA levels (Hunt et al., 2010). In contrast, in *C.* gigas, a negative correlation was observed between gbM and the mRNA expression levels (Riviere et al., 2013). Thus, associations between gbM and transcription may exhibit species-specific features and heavily depend on the density of methylation (Riviere, 2014). Indeed, here, we observed that in the genus *Trichinella*, genes heavily methylated exhibited a broad range of expression, whereas genes weakly methylated expressed at high levels. This contradicts the associations observed in other invertebrate species, suggesting more subtle roles and functional outcomes for this epigenetic mark in the genus *Trichinella*.

Characterization of stage-specific expressed genes that are developmentally regulated in the parasite life-cycle is an essential step toward understanding parasite biology and host-parasite interactions in the genus *Trichinella* (Liu et al., 2007, 2015; Ren et al., 2019). Yang and colleagues have characterized an NBL stage-specific expressed serine proteinase using monoclonal antibodies and proved its usage as major antigen in the early detection of *T. spiralis* infection (Yang et al., 2015). However, homologous E/S products may exhibit species-specific antibody responses, such as 53-kDa glycoprotein in mice with infection of five *Trichinella* species using recombinant antigens, suggesting the need to design species-specific antigens in immunodiagnosis in different host classes (Nagano et al., 2008). This observation is concordant with our findings that there are only three conserved SCOs of stage-specific expressed genes among these *Trichinella* species. Furthermore, in our recent publication, we revealed that epigenetic mechanism plays a role in regulating the expression of several stage-specific expressed genes, such as TP12446 (Liu et al., 2021), further demonstrating functional significance of these hyper-/hypo-methylated SCOs for the elucidation of parasite development and survival within the host.

Based on these findings, we further assessed the functional significance of SCOs with hyper-/hypo-methylated gene-bodies or promoters on transcriptome variation between the two clades of *Trichinella* species. We observed fam4516, an Ad stage-specific expressed SCO encoding a kinase-specific co-chaperone Cdc37, was highly expressed in the non-encapsulated clade. Functional roles of Cdc37 have been investigated in rabies virus, which could be proposed as therapeutic targets against rabies by directly affecting the life cycle of rabies virus and stability of viral P protein (Xu et al., 2016). The observed hypermethylation of gene-bodies of Cdc37 in the three species of non-encapsulated clade might provide potential DNA methylation markers preventing parasite invasion and survival. Further, we observed members of fam2698, which was annotated as a p53-like transcription factor, were hypomethylated in promoters with increased expression in ML stage in the non-encapsulated clade. Functions of p53 or p53-like transcription factors have been investigated in *Leishmania* spp., which has been mediating the apoptosis process of infected host macrophages and thus acting as one of the survival mechanisms of the parasites (Moshrefi et al., 2017). We speculate this higher expression of fam2698 in non-encapsulated clade may be associated with enhanced ability of parasite invasion and attack to adapt to their non-encapsulated phenotype in ML stage.

In the present study, we described the first comprehensively comparative epigenome study in the genus *Trichinella*. We observed SCOs with hyper-/hypo-methylated features in gene-bodies or promoters exhibited functional roles in gene regulation, especially for stage-specific expressed genes, which showed divergent features among those *Trichinella* species. By comparing the two clades of *Trichinella* in epigenomics and transcriptomics, we identified a panel of SCOs with altered methylation and expression, which were important for elucidating differential parasitology and epidemiology between the two clades of *Trichinella*. Our data provided here could be fully understanding the conservation and divergence of DNA methylation on differential adaptation and parasitism of these *Trichinella* species, which could lead to the development of new drugs by targeting the DNA methylation process for the treatment of trichinellosis.

## Data Availability Statement

Raw sequencing data were deposited in the GEO with the following accession numbers: GSE140382 for RNA-seq data, GSE139486 for WGBS data, and GSE169404 for WGS data.

## Ethics Statement

All experimental protocol involving animals have been reviewed and approved by the Ethical Committee of the Jilin University affiliated with the Provincial Animal Health Committee, Jilin Province, China (Ethical Clearance number IZ-2009-08).

## Author Contributions

FG and ML conceived the project. YF and FG analyzed and interpreted the high-throughput sequencing data. XL and BT undertook sample collection. XB and XW performed DNA extraction. XL and CL performed RNA extraction experiments. YL constructed high-throughput sequencing libraries, including WGS, WGBS, and RNA-seq. YF performed all of the bioinformatics analyses and was the main contributor in writing the manuscript. FG, ML, and YD critically reviewed the manuscript. All authors read and approved the manuscript for publication.

## Conflict of Interest

The authors declare that the research was conducted in the absence of any commercial or financial relationships that could be construed as a potential conflict of interest.

## Footnotes

1 https://www.bioinformatics.babraham.ac.uk/projects/fastqc/

2 http://www.microbesonline.org/fasttree/
