# Supplementary material for: Comparative Epigenomics Reveals Host Diversity of the Trichinella Epigenomes and Their Effects on Differential Parasitism
Source: Front Cell Dev Biol. 2021 Jun 11;9:681839. doi: 10.3389/fcell.2021.681839 (PMC8226246; doi:10.3389/fcell.2021.681839)
Supplement: Supplementary file 1 [file Table_1.docx]

**Supplementary Data Legends:**

**Supplementary Data S1.** 2708 single-copy orthologous genes of the twelve *Trichinella* species based on the protein sequence similarity.

**Supplementary Data S2.** Data generation for WGS, WGBS, and RNA-seq data.

**Supplementary Data S3.** Functional annotation of hypo- and hypermethylated genes in *T. pseudospiralis* in Ad stage.

**Supplementary Data S4.** Stage-specific expressed genes under hyper-/hypo-methylation in Ad and ML stages across the twelve *Trichinella* species. NA represents the homologous gene is not expressed in a stage-specific manner. Genes color-coded in green are conserved across the twelve *Trichinella* species.

**Supplementary Data S5.** Functional annotation of differential expressed genes in Ad and ML stages.

**Supplementary Figures:** See below


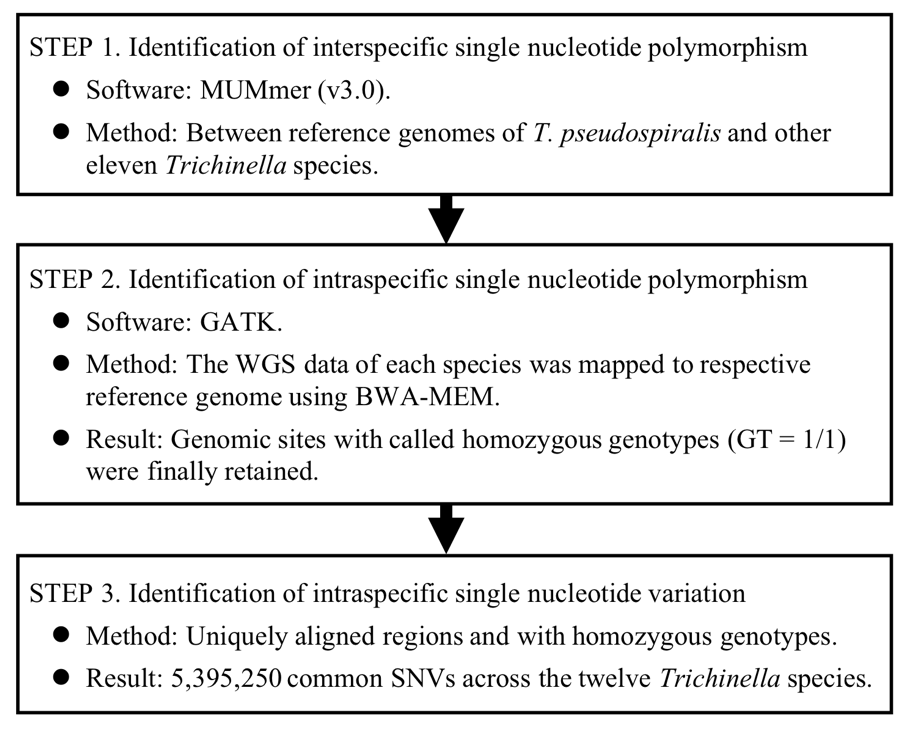


**Supplementary Fig. 1** Flowchart of single nucleotide variation identification process.


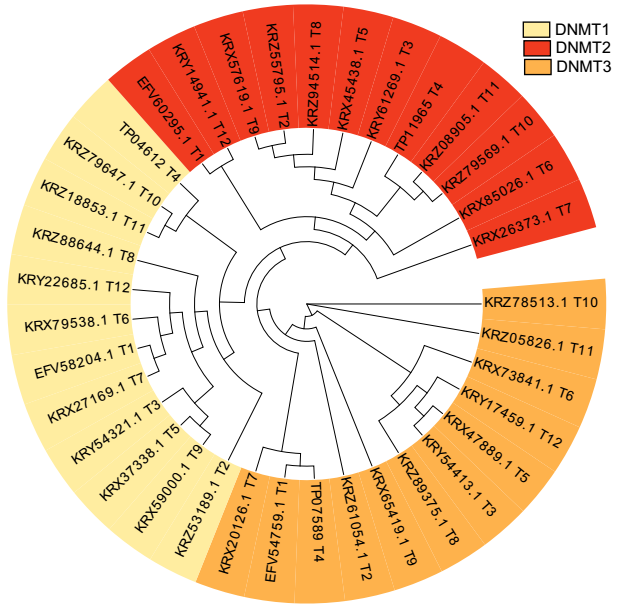


**Supplementary Fig. 2** Phylogenetic relationships of DNMTs across the twelve *Trichinella* species.


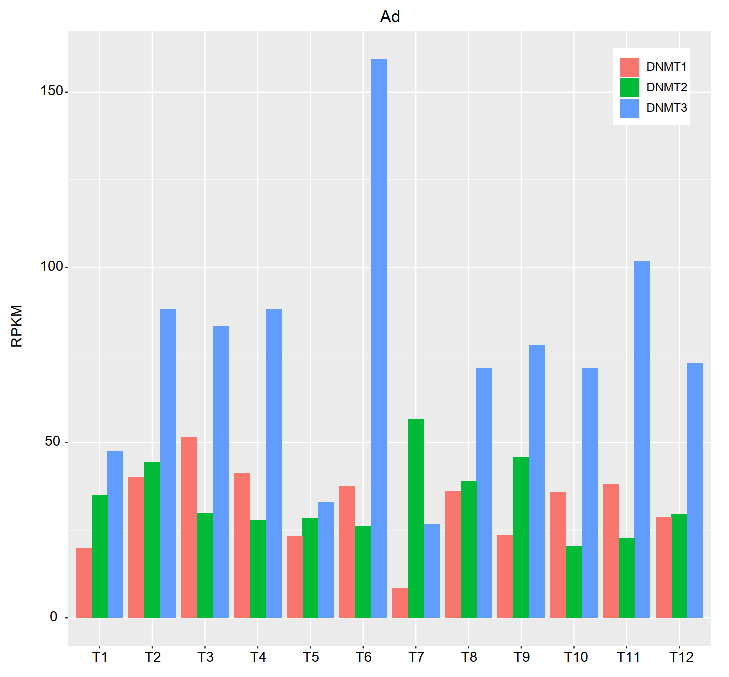

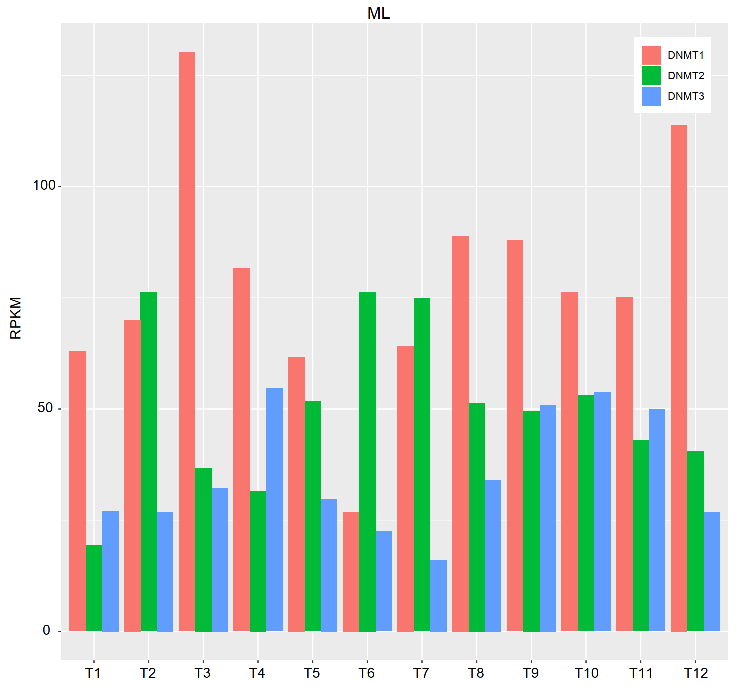

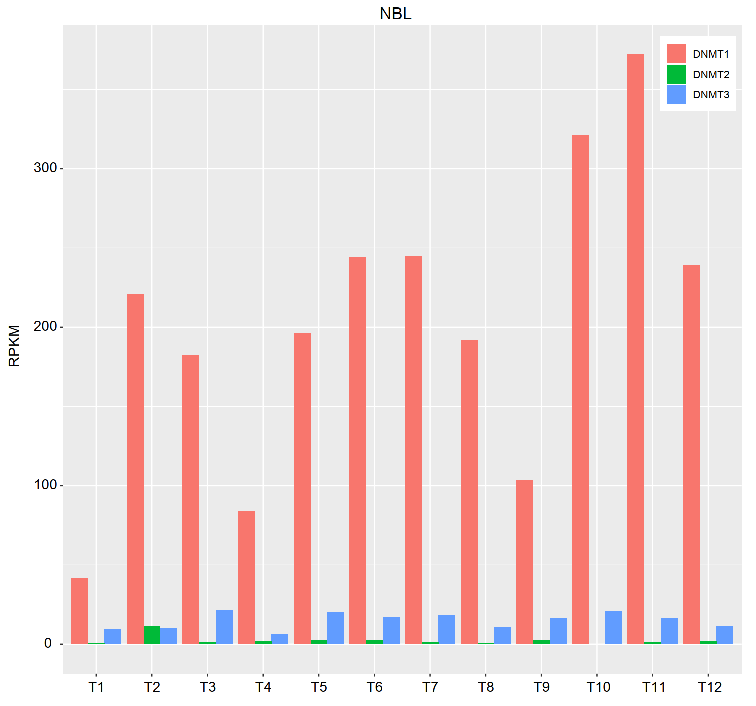


**Supplementary Fig. 3** RPKM values of the three DNMTs in the three life stages of the twelve *Trichinella* species.


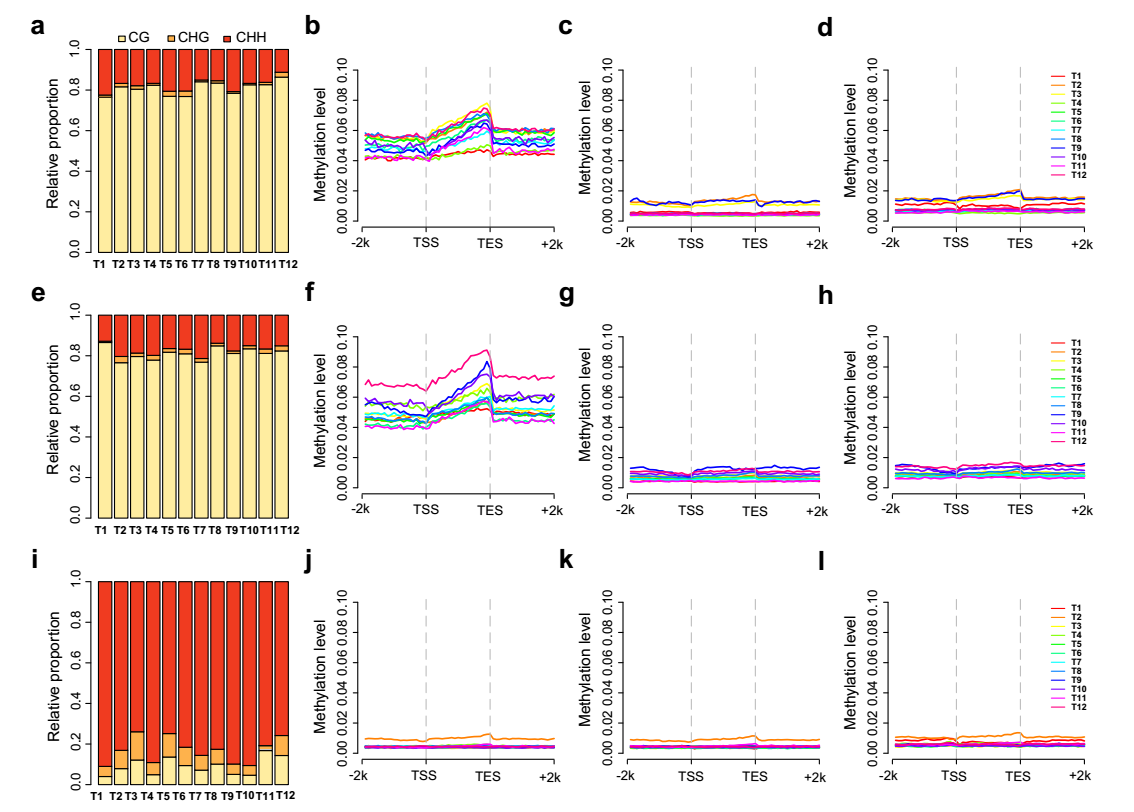


**Supplementary Fig. 4** DNA methylation patterns of the twelve *Trichinella* species. **(a, e, g)** The relative proportion of methylated CpGs (**a**), CHG (**e**), and CHH (**g**). Methylation profiles of methylated CpG sites (**b, f, j**), CHG sites (**c, g, k**) and CHH (**d, h, l**) around genic regions and 2-Kb flanking regions. 2-Kb region upstream and downstream of each gene was divided into 100 bp intervals. The gene region was divided into 20 intervals (5% per interval).


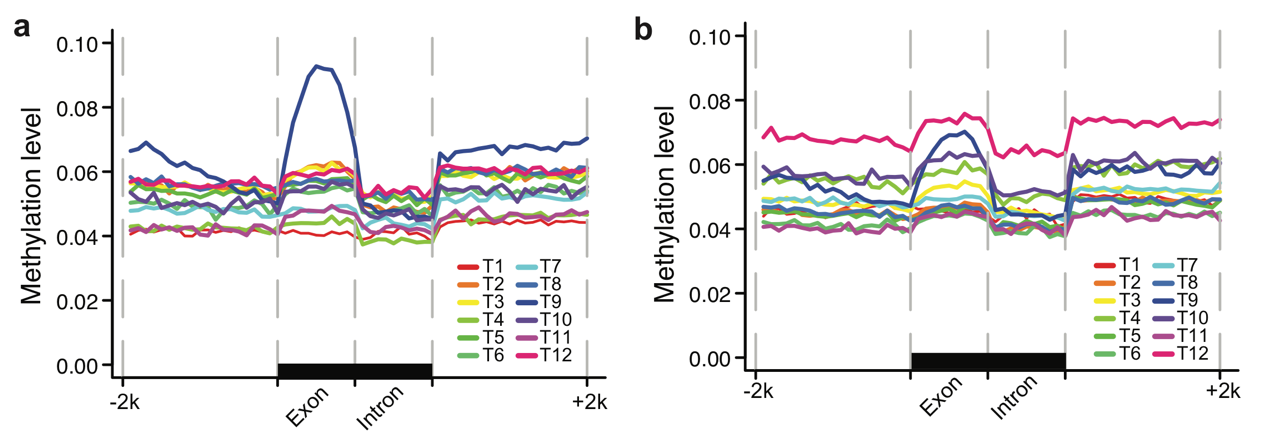


**Supplementary Fig. 5** CpG methylation levels of genic regions both in Ad (a) and ML (b) stages. 2-Kb region upstream and downstream of each gene was divided into 100 bp intervals. The gene region was divided into exons and introns, and each element was divided into 10 intervals (10% per interval).


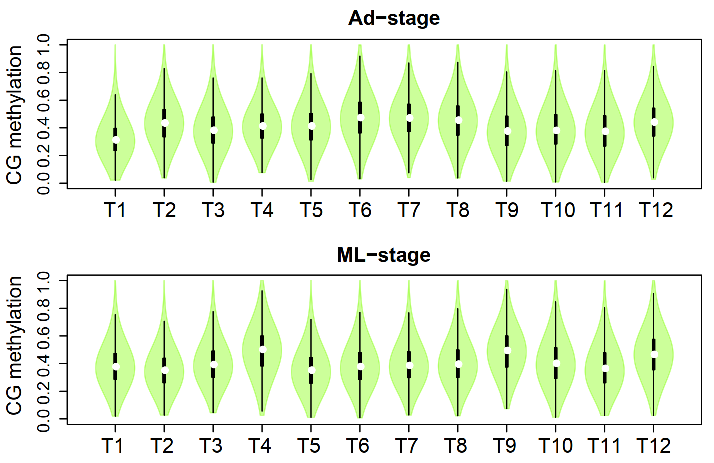


**Supplementary Fig. 6** CpG methylation levels of repeat regions both in Ad and ML stages.


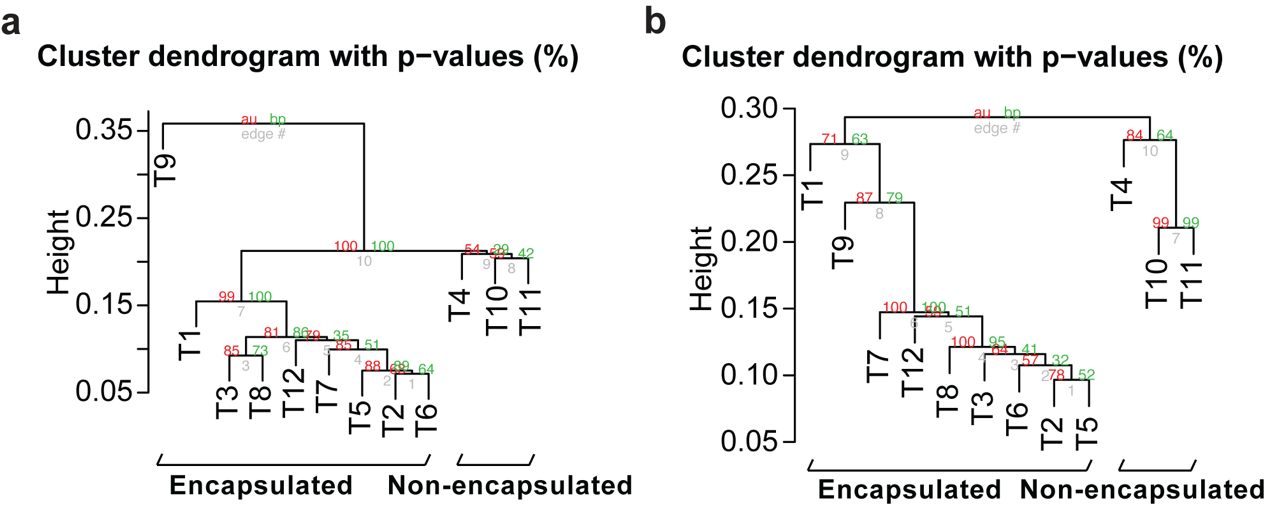


**Supplementary Fig. 7** Hierarchical clustering analysis based on DNA methylation levels of the gene-bodies (a) or promoters (b) of the 2708 SCOs in ML stage.


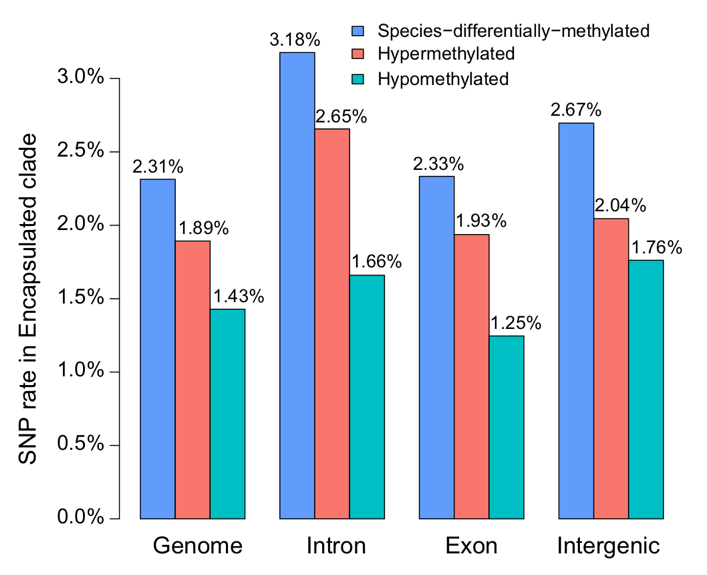


**Supplementary Fig. 8** SNP rate in hyper-, hypo-, and interspecies-differentially methylated regions among genome, intron, exon, and intergenic regions. As exemplified by the analysis between T1 and T7, here we observed differences between hyper- and hypo-methylated regions, and between hyper-methylated and interspecies-differentially-methylated regions were significant with a two-proportion z-test *P*-value < 0.001. Methylation levels of CpG sites higher than 0.3 was regarded as hyper-methylated sites, whereas methylation levels of CpG sites lower than 0.05 was regarded as hypo-methylated sites. The substitution rates of hyper- or hypo- methylated sites were performed around its upstream and downstream 80 bases.


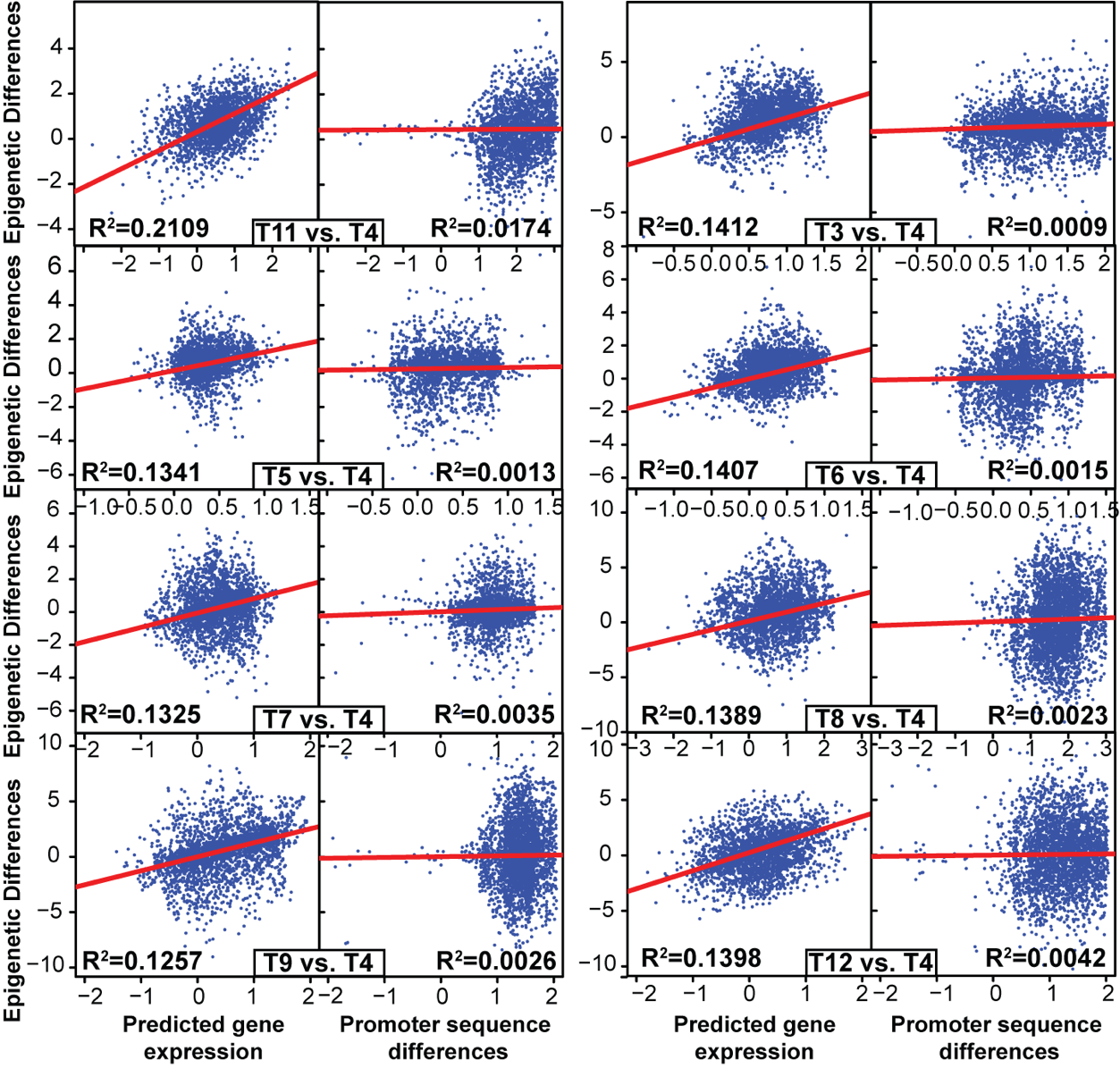


**Supplementary Fig. 9** Correlations among DNA methylation changes, gene expression alterations, and sequence differences in other eight *Trichinella* species (except for *T. spiralis*, *T. nativa*, and *T. papuae*) when comparing with reference species *T. pseudospiralis*.
